# Body Image Distress and Its Associations From an International Sample of Men and Women Across the Adult Life Span: Web-Based Survey Study

**DOI:** 10.2196/25329

**Published:** 2021-11-04

**Authors:** Alyssa Milton, Ashlea Hambleton, Anna Roberts, Tracey Davenport, Anna Flego, Jane Burns, Ian Hickie

**Affiliations:** 1 Brain and Mind Centre The University of Sydney Sydney Australia; 2 Sydney School of Medicine Faculty of Medicine and Health University of Sydney Camperdown Australia; 3 Project Synergy, InnoWell Pty Ltd Sydney Australia; 4 Inside Out Institute for Eating Disorders Charles Perkins Centre University of Sydney Sydney Australia; 5 The Movember Foundation, Australia East Melbourne Australia

**Keywords:** body image, mental health, well-being, web-based survey, sex differences, age

## Abstract

**Background:**

Previous research on body image distress mainly relied on samples that were small, generally homogeneous in age or sex, often limited to one geographical region, and were characterized by a lack of comprehensive analysis of multiple psychosocial domains. The research presented in this paper extends the international literature using the results of the web-based Global Health and Wellbeing Survey 2015. The survey included a large sample of both men and women aged ≥16 years from Australia, Canada, New Zealand, the United Kingdom, or the United States.

**Objective:**

The main objectives of this study are to examine body image distress across the adult life span (≥16 years) and sex and assess the association between body image distress and various psychosocial risk and protective factors.

**Methods:**

Data were extracted from the Global Health and Wellbeing Survey 2015, a web-based international self-report survey with 10,765 respondents, and compared with previous web-based surveys conducted in 2009 and 2012.

**Results:**

The body image distress of young Australians (aged 16-25 years) significantly rose by 33% from 2009 to 2015. In 2015, 75.19% (961/1278) of 16- to 25-year-old adults reported body image distress worldwide, and a decline in body image distress was noted with increasing age. More women reported higher levels of body image distress than men (1953/3338, 58.51% vs 853/2175, 39.22%). Sex, age, current dieting status, perception of weight, psychological distress, alcohol and other substance misuse, and well-being significantly explained 24% of the variance in body image distress in a linear regression (*F*_15,4966_=105.8; *P*<.001).

**Conclusions:**

This study demonstrates the significant interplay between body image distress and psychosocial factors across age and sex.

## Introduction

### Background

There is a clear need to understand and address body image distress, particularly when considering the increasing prevalence rates of body image distress worldwide [1-3] and the noted relationship between body image distress and mental ill-health [4]. Furthermore, there is scant large-scale international research examining body image across the adult life span [4] and from the perspectives of both men and women [5].

Body image is a multifaceted construct encompassing one’s body-related self-perceptions and self-attitudes, including thoughts, feelings, behaviors, and beliefs toward the body [6]. Research suggests that body image dissatisfaction occurs when there is a discrepancy between how an individual views their body (*actual body image*) and how they want it to be (*ideal body image*) [7,8]. Dissatisfaction, overevaluation, and preoccupation are considered as contributing factors to body image distress [9]. Current body ideals predominantly promote thinness for women and muscularity for men [10,11]. Given the difference between current body ideals and the actual body shape and size of most of the population, it is not surprising that many people view their bodies negatively and experience distress because of this negative self-perception [12].

### Body Image Distress Over Time

Body image is ranked as a top concern for young people [13]. Research suggests that the proportion of the population experiencing increased body image distress is increasing. It has been well documented that viewing appearance-focused media contributes to the development of body image concerns [14,15]. In the past 10 years, there has been a stark rise in multimedia platforms, such as Instagram, Snapchat, and TikTok. The imagery on social media is often filtered and edited in a way that promotes an unrealistic appearance ideal. Research has shown that social media use is associated with increased body image dissatisfaction [14] because it facilitates comparison, and appearance-related comments and praise are reinforced with likes, follows, and comments.

### Body Image, Age, and Sex

The association between weight status (as measured by BMI) and body image dissatisfaction has been previously explored [16]. Research has demonstrated that increased BMI is associated with greater body image dissatisfaction in college students [17], help-seeking adults [18], adolescent men [19], and nonclinical samples of adult women [20]. Risk of body dissatisfaction is not restricted to individuals with higher BMI; adolescent women with either a healthy or an overweight BMI experience higher levels of body dissatisfaction, whereas underweight women have the highest levels of body satisfaction [19]. Similarly, in a sample of middle-aged women, 70% of participants reported a desire to be thinner, despite most being considered normal weight [21]. Overall, these findings fit the current cultural narrative that a thin body is both desirable and idealized in Western societies [22].

Although the research has mainly focused on young women, some studies indicate that body image concerns are pervasive across the adult life span [12] for both sexes [5]. The picture appears to be more complex for men [23]. For example, adolescent men tend to be equally divided between wanting to lose weight (predominantly high body fat) and gain weight (muscle mass) [5,24]. As men move into adulthood, there is an increase in the desire to lose weight [5]. Within cohorts of middle-aged women, only 11% of participants endorsed being satisfied with their bodies [25]. Women’s dissatisfaction with their bodies appears relatively stable across the adult life span [8,12]. However, some research suggests that the impact of body image on an individual’s self-esteem and self-concept may diminish over time [26]. Furthermore, there is more tolerance in what body sizes are considered acceptable with increasing age [4,27]. However, overall, body image research looking at age, sex, and weight is fragmented, and a comprehensive picture is lacking.

### Body Image Distress and Psychopathology

Body image dissatisfaction in childhood and early adolescence can predict adverse health outcomes in later life, including engaging in dangerous weight control behaviors and general psychological distress [28]. As highlighted above, research with adolescents is much more extensive than with their adult counterparts, with several studies demonstrating an association between body dissatisfaction and anxiety [29-33], depression [29,30,34-37], self-harm [38-42], and low self-esteem [28,36,37].

Research has reported that body image dissatisfaction is associated with higher levels of depression, anxiety, disordered eating [43-45], and distress [46]. Furthermore, research has identified associations between body image with other aspects of health, such as tobacco smoking [47], alcohol misuse [48], poor self-esteem [18,49,50], and poor mental and physical health–related quality of life [46].

Conversely, optimism, positive affect, self-compassion, life satisfaction, and subjective happiness [51-61] are associated with positive body image. Social well-being has also been reported to play a part in both positive and negative body image, particularly in adolescents [59,62-64]. For example, Bearman et al [63] observed higher levels of body dissatisfaction in girls and boys who had deficits in their social support from parents and peers. Meanwhile, individuals with more supportive parental relationships have reported higher body image satisfaction [62].

### Current Research

Previous research exploring body image distress mainly relied on small samples that were generally homogeneous in age or sex, lacked a comprehensive analysis of multiple psychosocial domains, and were limited to one geographical region. This study extends the international literature using the results of the Global Health and Wellbeing Survey 2015, a large web-based sample of both men and women (aged ≥16 years) from Australia, Canada, New Zealand, the United Kingdom, or the United States. Additional data sources included the *headspace* web-based Community Youth Survey (2009) and the Young and Well First National Survey on the web (2012).

This study has 3 main aims, including the assessment of (1) the changes in body image distress over time (between 2009 and 2015) for young people aged 16 to 25 years; (2) the associations between weight range (BMI), dieting status, and perceived body image distress by sex and age group; and (3) the association of various demographic, health, and well-being factors with body image distress or preoccupation.

## Methods

### Participants

Participants were a voluntary community sample of men and women (aged ≥16 years) who reported that they had lived in 1 of the 5 target countries (Australia, Canada, New Zealand, the United Kingdom, or the United States) for the best part of the past 12 months. A total of 16,510 people reviewed the consent and eligibility screen. Of the 16,510 people, the total eligible sample was 10,765 (65.2%) respondents. Of those excluded, 26.2% (4326/16,510) did not consent to participate, and 4.3% (710/16,510) were younger than 16 years.

### Procedures and Recruitment

The primary study received institutional ethics approval from The University of Sydney Human Research Ethics Committee (protocol 2015/412). All procedures complied with the ethical standards of the relevant national and institutional committees on human ethics and the Helsinki Declaration of 1975, as revised in 2008.

The survey was hosted on the internet from July 1, 2015, to December 11, 2015. For optimizing recruitment in the 5 target countries, the following strategies were used: both paid and free advertising across multiple social media channels such as Facebook, Twitter, and YouTube for survey dissemination [65]; layering of recruitment messages [66]; passive web-based snowballing via social media to spread study information through sharing, liking, and tweeting [65,67]; and traditional snowballing [68]. Targeted recruitment based on age, sex, and region was carried out through paid advertising channels to maximize responses from groups hard to reach. Respondents consented on the website and were informed that their responses were confidential, nonidentifiable, and that they could cease participation at any time. Participants were informed that the survey would take between 20 and 45 minutes to complete depending on participant answers and the survey skip pattern. Any respondents indicating psychological distress or problematic alcohol or substance use in their responses were provided with the contact details of local support lines.

### Materials

Items in this substudy were extracted from the Global Health and Wellbeing Survey 2015 [69], and where items could be compared, from the *headspace* web-based Community Youth Survey (2009) and the Young and Well First National Survey on the web (2012)*.* Areas of interest specific to this substudy are described in the following sections.

#### Demographics

The respondents first provided their sex (men and women) and age (16-25 years, 26-49 years, or ≥50 years).

#### Weight, Body Image, and Eating Behaviors

BMI was determined by asking respondents their weight (kg or lb) and height (meters or feet and inches). Respondents with a BMI less than 18.5 were classified as *underweight*, a BMI between 18.5 and 24.9 as *healthy weight*, a BMI between 25.0 and 29.9 as *overweight*, and a BMI higher than 30.0 as *obese*.

Current dieting status was determined by the question “Are you currently dieting?” adapted from Blashill and Wilhelm [70]; this item provided 3 response options (“yes, to lose weight”; “yes, to gain weight”; and “no”).

For assessing body image attitudes, respondents were asked to self-evaluate the importance of weight and shape for them over the past 3 months: “How much has your shape influenced how you feel as a person?” This question was answered on a 6-point Likert scale ranging from *not at all* to *a great deal*.

For assessing body image distress or preoccupation, respondents were asked, “Do you get very distressed or preoccupied by any specific aspect of your physical appearance?” using a dichotomous *yes* or *no* response option. If respondents indicated distress or preoccupation, a follow-up question asked which areas of their body they were concerned about, such as *facial features*, *arms or legs*, and *weight* [71]. This body image distress item was also asked in the web-based *headspace* Community Youth Survey in 2009 and the Young and Well National Survey on the web in 2012 with 16- to 25-year-old adults [71]. These data were used in this research for longitudinal cohort comparisons.

#### Mental Health and Well-being

Physical activity was measured by the International Physical Activity Questionnaire short form, which classifies individuals into 1 of 3 levels of physical activity (*inactive* vs *minimally active* vs *health-enhancing physical activity*) [72].

Current psychological distress was measured using the 10-item Kessler Psychological Distress Scale (K10) [73]. Total scores were grouped into 4 levels of psychological distress (10-15=low, 16-21=moderate, 22-29=high, and 30-50=very high) [74].

Respondents’ levels of suicidal thoughts and behaviors in the past 12 months were measured using the 5-item suicidal thoughts and acts subscale from the Psychiatric Symptom Frequency Scale [75].

The likelihood of alcohol or other substance misuse was calculated using 2 items. If respondents positively endorsed one of either item, “...recently thought that you should cut down...” or “...another person suggested you should cut down...,” they were categorized as having a *possible* alcohol or other substance misuse. Endorsement of both items resulted in *probable* alcohol or other substance misuse. Endorsement of neither item resulted in being placed in the *not likely* category.

*Days out of role* was extracted from the Brief Disability Questionnaire [76] to investigate functioning. The 7-item Personal Well-being Index [77] was used to assess subjective well-being.

Happiness was measured by the 4-item Oxford Happiness Questionnaire [78], and resilience was measured by the 4-item Brief Resilience Coping Scale [79].

Perceived social support and conflict in close relationships were measured by the 5-item Schuster Social Support and Conflict Scale [80].

### Analysis

Survey data were prepared and analyzed using IBM SPSS Statistics for Windows, Version 22.0 (IBM Corp, 2013). For addressing aim 1, body image distress data in 2009 and 2012 were compared with 2015 data using a one-sample two-tailed *t* test. To address aim 2, descriptive and frequency statistics were used to describe all weight, dieting, and body image items by basic demographics (sex and age). Chi-square and analysis of variance tests were completed to compare items by sex and age. For addressing aim 3, an initial scoping analysis using Pearson product-moment *r* correlations [81] was conducted to independently assess the strength of the relationship between body image distress and these health and well-being items (healthy weight; *no* vs *yes* based on BMI data), current dieting status (*no* vs *yes*), perception of weight (*about the right weight* vs *all others*), physical activity (International Physical Activity Questionnaire short form), psychological distress (K10), suicidal ideation (Psychiatric Symptom Frequency Scale), alcohol and/or other substance misuse, days out of role, well-being (Personal Well-being Index), happiness (Oxford Happiness Questionnaire), resilience (Brief Resilience Coping Scale), intimate bonds (Intimate Bond Measure), and social support (Social Support and Conflict Scale). A subsequent linear regression analysis was conducted to determine which of the health and well-being items significantly explained variance in body image distress when considered together while controlling for sex and age.

## Results

### Respondent Participation Rates and Characteristics

Most of the eligible respondents were women (6464/10,765, 60.05%). Of the 10,753 respondents, 2874 (26.73%) were aged between 16 and 25 years, 2879 (26.77%) were aged between 26 and 49 years, and 5000 (46.49%) were aged ≥50 years. A breakdown of demographics by country, age, and sex is presented in Table 1. Further demographic details can be found in related publications [82,83].

**Table 1:** Participant demographics by country, age, and sex (N=10,765).

Characteristics			Total		Australia		Canada		New Zealand		United Kingdom		United States
Country, n (%)			10,765 (100)		3349 (31.11)		1888 (17.54)		1752 (16.27)		1938 (18)		1838 (17.07)
**Sex, n (%)**													
	Women	6464 (60.05)		2067 (61.72)		1097 (58.1)		1055 (60.22)		1176 (60.68)		1069 (58.16)	
	Men	4301 (39.95)		1282 (38.28)		791 (41.89)		697 (39.78)		762 (39.32)		769 (41.84)	
Age (years), mean (SD)			44.37 (19.68)		42.44 (18.71)		52.46 (17.84)		39.65 (19.51)		42.57 (19.40)		45.97 (21.07)

### Main Findings

#### Aim 1: Changes in Body Image Distress Over Time Across the Life Span

Of those young Australians (aged 16-25 years) who completed the 2009 (*headspace* web-based Community Youth Survey), 2012 (Young and Well First National Survey on the web), or 2015 (Global Health and Wellbeing Survey) surveys, self-reported body image distress rose by 33% from 2009 to 2015 (419/949, 44.2% reported distress in 2009; 1158/1731, 66.89% in 2012; and 300/388, 77.32% in 2015). The mean difference was significant across both the 2009 and 2012 time points (2009 vs 2015: *t*_387_=15.56, *P*<.001; 2012 vs 2015: *t*_387_=4.90, *P*<.001).

#### Aim 2: Body Image, BMI, and Dieting by Age and Sex

Table 2 displays frequency statistics for measures of body image items, BMI, and current dieting status by age band and sex from the Global Health and Wellbeing Survey 2015. Significance tests comparing items across sex and age are presented in Multimedia Appendix 1. Approximately half of all respondents (2806/5513, 50.89%) reported feeling very distressed or preoccupied with their body image. Women reported higher levels of distress related to body image than men (1953/3338, 58.51% vs 853/2175, 39.22%). Respondents aged 16-25 years showed higher levels of body image distress than all other age groups, at 75.19% (961/1278), with distress decreasing as age increased.

**Table 2:** Frequency statistics for measures of body image item by age and sex (maximum N=5517).

Body image item		Sex			Age bands (years)				Full sample
		Men	Women	16-25		26-49	≥50		
**Body image distress or preoccupation, n (%)**		2175 (39.45)	3338 (60.55)	1278 (23.18)		1502 (27.24)	2733 (49.57)	5513 (100)	
	Yes	853 (39.22)	1953 (58.51)	961 (75.19)		837 (55.73)	1008 (36.88)	2806 (50.89)	
	No	1322 (60.78)	1385 (41.49)	317 (24.8)		665 (44.27)	1725 (63.12)	2707 (49.1)	
**How much does weight or shape influence how you think of yourself as a person**		2169 (39.47)	3327 (60.53)	1275 (23.19)		1497 (27.24)	2724 (49.56)	5496 (100)	
	1=not at all, n (%)	510 (23.51)	425 (12.77)	130 (10.17)		181 (12.09)	624 (22.91)	935 (17.01)	
	6=a great deal, n (%)	256 (11.8)	771 (23.17)	330 (25.88)		321 (21.44)	376 (13.8)	1027 (18.69)	
	Score, median (IQR)	3 (2-4)	4 (2-5)	4 (3-6)		4 (2-5)	3 (2-5)	4 (2-5)	
**BMI, n (%)**		2115 (39.32)	3264 (60.68)	1248 (23.2)		1464 (27.22)	2667 (49.58)	5379 (100)	
	Underweight	30 (1.42)	141 (4.32)	97 (7.77)		39 (2.67)	35 (1.31)	171 (3.18)	
	Healthy weight	671 (31.73)	1344 (41.19)	721 (57.77)		580 (39.64)	714 (26.77)	2015 (37.47)	
	Overweight	791 (37.39)	836 (25.62)	232 (18.59)		438 (29.94)	957 (35.88)	1627 (30.25)	
	Obese	623 (29.46)	842 (28.87)	198 (15.87)		406 (27.75)	961 (36.03)	1565 (29.09)	
**Self-evaluation of weight, n (%)**		2176 (39.46)	3339 (60.54)	1279 (23.19)		1503 (27.25)	2733 (49.56)	5515 (100)	
	Very underweight	25 (1.15)	19 (0.56)	16 (1.25)		9 (0.59)	19 (0.69)	44 (0.79)	
	Slightly underweight	154 (7.08)	143 (4.28)	129 (10.09)		74 (4.92)	94 (3.44)	297 (5.39)	
	About the right weight	646 (29.69)	1057 (31.66)	574 (44.88)		522 (34.73)	607 (22.21)	1703 (30.88)	
	Slightly overweight	1000 (45.96)	1348 (40.37)	429 (33.54)		624 (41.52)	1295 (47.38)	2348 (42.57)	
	Very overweight	351 (16.13)	772 (23.12)	131 (10.24)		274 (18.23)	718 (26.27)	1123 (20.36)	
**Currently dieting, n (%)**		2178 (39.48)	3339 (60.52)	1278 (23.16)		1503 (27.24)	2736 (49.59)	5517 (100)	
	Yes, to lose weight	427 (19.61)	868 (25.99)	268 (20.97)		374 (24.88)	653 (23.87)	1295 (23.47)	
	Yes, to gain weight	49 (2.25)	27 (0.81)	38 (2.97)		17 (1.13)	21 (0.77)	76 (1.38)	
	No	1702 (78.15)	2444 (73.20)	972 (76.06)		1112 (73.99)	2062 (75.37)	4146 (75.15)	

Only 17.01% (935/5496) of respondents in the full sample indicated that their weight and shape did not influence how they thought of themselves as a person. Women (425/3327, 12.77%) and the younger age bands (16-25 years: 130/1275, 10.19%; 25-44 years: 181/1497, 12.09%) were significantly less likely to endorse that their weight or shape did not influence their self-perception (*P*<.001).

Although 57.77% (721/1248) of young people (16-25 years) were in the healthy BMI range, fewer (574/1279, 44.88%) considered themselves about the right weight. This pattern was repeated in women, of whom 41.19% (1344/3264) were in the healthy BMI range, but fewer (1057/3339, 31.66%) endorsed that they were about the right weight. The percentage of men who were in a healthy BMI range was 31.73% (671/2115), which reflected the rates of men who felt they were about the right weight (646/2176, 29.69%). In the older age brackets, more participants were in the obese BMI category (26-49 years: 406/1464, 27.75%; ≥50 years: 961/2667, 36.03%) than those who felt that they were very overweight (26-49 years: 274/1503, 18.23%; ≥50 years: 718/2733, 26.27%). Across the full sample, 23.47% (1295/5517) of all participants were currently dieting to lose weight, and 1.38% (76/5517) were currently dieting to gain weight. Across ages, not engaging in any dieting was relatively consistent (between 1112/1503, 73.99% and 972/1278, 76.06%). Women reported they were dieting to lose weight significantly more frequently (868/3339, 25.99%) than men (427/2178, 19.61%; *P*<.001).

For addressing aim 3, a series of linear regressions were conducted to examine the relationship between body image distress or preoccupation and health and well-being items. Table 3 presents the full sample results (Multimedia Appendix 2 for each age group). Individual Pearson product-moment *r* correlations for each health and well-being item by body image distress are presented in Multimedia Appendix 3. The regression model using the full sample significantly accounted for 24% of the variance in body image distress or preoccupation (*F*_15,4966_=105.8; *P*<.001; *R*^2^_adj_=0.24). After controlling for sex (β=.12; *P*<.001) and age (β=−0.24; *P*<.001), 5 variables significantly explained model variance. This included current dieting status (β=.13; *P*<.001), perception of weight (β=.09; *P*<.001), psychological distress (β=.21; *P*<.001), alcohol and/or other substance misuse (β=.04; *P*<.001), and well-being (β=−0.07; *P*<.001). Specifically, respondents who were currently dieting reported body image distress or preoccupation more frequently. Those who did not report that they were *about the right weight* reported higher psychological distress and had a higher likelihood of problematic alcohol or other substance use and higher body image distress or preoccupation. Participants with higher personal well-being scores reported lower levels of body image distress or preoccupation.

**Table 3:** Linear regression of body image distress (*F*_15,4966_=105.8; *P*<.001; *R*^2^_adj_=0.24).

Variable		*t* (*df*)^a^	*P* value	β (95% CI)
**Body image distress or preoccupation**				
	Healthy weight (no vs yes based on BMI)	0.04	.97	.00 (−0.03 to 0.03)
	Current dieting (no vs yes)	10.20	<.001	.13 (0.12 to 0.18)
	Perception of weight (about the right weight vs not)	6.06	<.001	.09 (0.07 to 0.13)
	Physical activity (IPAQ^b^)	−0.03	.97	.00 (−0.02 to 0.02)
	Psychological distress (K10^c^)	9.45	<.001	.21 (0.01 to 0.01)
	Suicidal ideation (PSFS^d^)	0.38	.71	.01 (−0.03 to 0.04)
	Alcohol and/or other substance misuse	2.82	<.001	.04 (0.01 to 0.04)
	Days out of role	−1.42	.16	−0.02 (−0.01 to 0.00)
	Well-being (PWI^e^)	−3.37	<.001	−0.07 (0.00 to 0.00)
	Happiness (OHQ^f^)	−0.40	.69	−0.01 (−0.01 to 0.00)
	Resilience (BRCS^g^)	0.84	.40	.01 (0.00 to 0.01)
	Social support (SSCS^h^)	−1.39	.16	−0.02 (−0.01 to 0.00)
	Intimate bonds (IBM^i^)	−0.57	.57	−0.01 (0.00 to 0.00)
	Sex	9.46	<.001	.12 (0.10 to 0.15)
	Age	−16.57	<.001	−0.24 (−0.01 to −0.01)

^a^*df*=15,4966

^b^IPAQ: International Physical Activity Questionnaire short form.

^c^K10: 10-item Kessler Psychological Distress Scale.

^d^PSFS: Psychiatric Symptom Frequency Scale.

^e^PWI: Personal Well-being Index.

^f^OHQ: Oxford Happiness Questionnaire.

^g^BRCS: Brief Resilience Coping Scale.

^h^SSCS: Schuster Social Support and Conflict Scale.

^i^IBM: Intimate Bond Measure.

When analyzed separately by age group (Multimedia Appendix 2), sex, current dieting status, perception of weight, and psychological distress consistently explained model variance across all age groups. Variation was found for happiness, alcohol or other substance misuse, and well-being items. Specifically, lower happiness also explained body image distress (β=−0.16; *P*=.003) in young people (aged 16-25 years). For those aged 26 to 49 years, alcohol and/or other substance misuse remained an item that explained body image distress (β=.07; *P*=.008). Conversely, for the ≥50 years age group, lower well-being continued to explain body image distress (β=−0.10; *P*=.001) variance.

## Discussion

### Principal Findings

To our knowledge, this is the largest international study to examine body image distress—and other related factors, including self-reported and perceived weight range and dieting status—across time, age, and sex. Our findings show that body image distress has become a highly prevalent issue by 2015. Of concern, considerable levels of body image distress were present in women and young people, and multiple psychosocial risk factors were associated with this distress.

One of our key findings comes from the cross-sectional longitudinal Australian data. Self-reported body image distress in young people aged 16 to 25 years increased from 44.2% (414/949) of those surveyed in 2009 to three-quarters (961/1278, 75.19%) in 2015. This finding is consistent with the increasing prevalence rates of body image distress in countries such as the United States [1]. Furthermore, although there are some suggestions in the literature that concern regarding body image has increased in Australia [84-86], this is the first known study to report changes across these 3 time points using web-based samples. Our data indicate that the issue is much more prevalent. This increasing prevalence of body image distress corresponds with the rise of social media. During the time frame of the survey, Instagram was launched in 2010, Snapchat was released in 2011, and TikTok was released in 2016. As photographs and videos are central to the use of these platforms, and previous research has shown an association between body image distress and social media use, perhaps this increased level of distress has occurred in parallel with the rise of social media [14,15].

This rise in prevalence is particularly concerning, given our findings that body image distress was associated with increased levels of current dieting, poorer self-perception of weight***,*** higher psychological distress, increased alcohol or other substance misuse, and poorer personal well-being. This is consistent with previous research where higher body image dissatisfaction directly correlated with poor mental health–related quality of life and psychosocial functioning [17]. Furthermore, literature examining body appreciation has reported associations with greater subjective happiness [56] and fewer days of feeling mentally or physically unhealthy [87]. It is unclear whether these factors are precipitating factors or consequences of body image distress. As 24% of the variability in body image distress was accounted for by these factors, future research could endeavor to explore what other factors are potentially missing from this model that also contribute to distress, such as social media use or history of disordered eating. Overall, when considering the rise of body image distress and its link to poorer psychosocial outcomes, a sharper focus on this area is needed.

Another important finding was that in our 2015 international sample, more than half of the participants’ BMI in the overweight or obese range (59.34%), with men reporting higher levels of obesity than women. This in itself is concerning, as obesity is considered one of the greatest health epidemics worldwide [88-91]. Furthermore, our findings demonstrated a notable sex difference concerning how men and women perceive their weight compared with their self-reported weight as measured by BMI. Specifically, despite a higher percentage of men having an overweight or obese BMI, more women (772/3339, 23.12%) considered themselves to be very overweight than men (351/2176, 16.13%). Although more women in this sample were in a healthy weight range (women: 1344/3264, 41.19%; men: 671/2115, 31.73%), only one-third of women believed they were *about the right weight*. This is consistent with data from previous studies demonstrating that women are more likely to perceive themselves as overweight compared with men [35,92-95]. In the literature, possible factors that may contribute to this discrepancy include self-esteem [96], sociocultural influences, and expectations [97-99].

Interventions in this area are relatively unexplored, particularly those targeting both men and women. Evidence-based interventions include self-monitoring, cognitive restructuring, exposure exercises, fitness training, mirror work [100], media literacy, self-esteem enhancement, and psychoeducation. However, these interventions only achieve minor improvements in body image [101,102]. Interventions with a greater focus on stress management training, cognitions, and negative body image causes appear to be more effective [101]. There is some evidence that self-compassion training can be beneficial for weight loss, nutrition behaviors, eating behaviors, and body image [103]. This training focuses on promoting self-worth, creating a more positive body image, and decreasing body dissatisfaction, and may be the way forward to improve health outcomes in distressed individuals.

These discrepancies between actual and perceived weight were not only a function of sex but also of age. Our 2015 survey results indicated that 57.77% of young people were in the healthy BMI weight range—the highest endorsement across all age groups. Despite this, three-quarters of young people reported body image distress. Again, this was the highest endorsement across all age groups. As participants aged, their BMI increased—with far more in the overweight and obese categories. However, the rate of body image distress declined as participants aged, as did the influence of weight or shape on how they viewed themselves as a person. Previous literature supports this phenomenon [104-107]. This change could be attributed to a shift in body comparisons with age-appropriate peers [8], less cultural fat phobia [105-107], or a focus on body function rather than body appearance [108].

Furthermore, research has theorized that people’s preoccupation and desire to change their body weight via dieting behavior becomes less salient with age [26]. Interestingly, our survey results demonstrated that approximately three-quarters of people reported they were not dieting, and this proportion remained relatively stable across each age group. Thus, although body image distress decreases with age, in line with the Webster and Tiggemann study [26], our contradictory finding is that dieting behaviors remain relatively consistent. Further research is needed to examine whether this is explained by the changes in people’s reasons for dieting as they age. For example, older people might be dieting for health or medical reasons rather than because of their body image concerns.

### Implications for Policy and Practice

This study supports the clear link between body image distress and poorer health and well-being [28]. Prevalence rates of body image dissatisfaction have increased worldwide in the past 30 years [109]. Our research shows that this prevalence is 3 in 4 young people when using a web-based survey methodology. These are compelling statistics. In 2019, the Australian government invested US $146 million into the prevention, detection, assessment, and treatment of eating disorders [110]. Although this is timely, our findings on the inverse relationship between individual distress and psychosocial outcomes make a strong case for the need for prevention and early intervention before eating disorders emerge. This may include more comprehensive assessment when accessing health services and the use of health information technologies to improve support services [111]. Given our findings, such interventions may benefit from targeting across sex and age.

Previously, body image distress was thought to result from the discrepancy between actual and perceived body image. However, our results suggest that the rates of body image distress within some age groups far exceed the proportion of the population who experience a discrepancy, indicative of other factors contributing to distress. For example, research is needed to determine whether these results are related to the considerable increases in the use and availability of social media in the past decade [14]. Exposure to social media, particularly in an individual’s formative years, could have a considerable impact on a young person’s sense of self, quality of life, and body image than is currently known. Some studies have demonstrated that the use of highly visual social media such as Instagram or Snapchat is linked to upward social comparison and internalization of symptoms of body dysmorphia, resulting in increased body image distress [112-115]. The more time spent on social media, the more significant the body image concerns [116-118]. Photograph-based activity (eg, posting photographs and viewing or making comments on others’ photographs) is particularly salient in contributing to body dissatisfaction and disordered eating [14]. As the data in this study were from a web-based sample recruited using social media channels, the results may reflect the experience of body image distress of a web-based population, who may be more likely to be using other platforms such as Instagram.

Body image distress and dieting behaviors are well understood to be risk factors for disordered eating and the development of an eating disorder. Our results indicate a relationship between dieting behavior, psychological distress, and the self-perception of weight, in addition to alcohol and/or other substance use and well-being impact on body image distress. The triangulation of items such as those used in this survey (BMI vs distress vs dieting status, or BMI vs distress vs perceptions of weight) may be beneficial as a brief screening tool. Given the burden of completing lengthy psychometrics, how these brief screeners compare with lengthier eating disorder questionnaires should be explored.

### Strengths and Limitations

A key strength of this study is that it is one of the largest samples to date, providing data on weight, perceptions of weight, dieting status, body image distress, and health and well-being. As outlined in the main report’s executive summary [69], a key limitation of the research is the nonepidemiological nature of the web-based research; targeting efforts were made for age, sex, and by country to address this. Although most individuals in the participating countries have widespread internet access [119], this study will also yield some level of internet bias, in that those who do not frequently access the internet or social media recruitment websites may not have participated. However, a major advantage to using a web-based surveying methodology is that previous research has found that it is associated with increased disclosure of sensitive information [120], such as the items asked in this survey. There is also the possibility of avidity bias occurring, as those with a greater interest in the subject may be more likely to participate [121]. However, overall, this research remains highly relevant, as it is the interactions between variables, not merely the statistical frequencies, that generate meaningful information. Furthermore, as we move further into the 21st century, web-based questionnaires may become more the norm than the exception.

Where possible, measures that have been tested for reliability and validity across general populations worldwide were used. For example, the K10 is the standard tool used to measure distress in Australia’s National Survey of Mental Health and Wellbeing (Burgess et al [122]) and is used widely in international studies. The BMI [121] is the most recommended and widely used tool for classifying weight range in adults [123]. However, the use of BMI is limited and has several deficiencies as a measure of obesity [124]. BMI is not a reliable reflection of health status, does not accurately reflect changes that occur with age, cannot account for muscle mass, and is a poor indicator of body fat percentage [123].

Furthermore, the participants were asked to self-report their height and weight. Responses may have been subject to bias as BMI is often calculated with overestimated height and underestimated weight data [125]. Furthermore, owing to the breadth and sheer size of the Global Health and Wellbeing Survey 2015, validated measures for eating disorders such as Eating Disorder Inventory, third edition [126] and the Eating Attitudes Test [127] were not viable to use. Instead, we asked brief questions (all adapted from established literature) on areas such as dieting status, perceptions of weight, and body image distress. Overall, the results are still meaningful. Although they may not be fully representative of the populations with eating disorders, they demonstrate a clear link between body image distress and health and well-being concerns.

### Conclusions

This research demonstrates the significant interplay between body image distress and psychosocial risk factors, including currently dieting, worse perceptions of weight, elevated psychological distress, increased alcohol or other substance misuse, and poorer personal well-being. Considering that an increasing number of young people are experiencing body image distress, body image should be closely monitored, given its association with poorer health outcomes. Further research into tailored intervention and prevention strategies for those experiencing any level of body image distress, obesity, eating disorders, and other health-related concerns is needed.

## Appendix

Multimedia Appendix 1 Frequency statistics, chi-square, and analysis of variance comparing body image items by age and sex.

Multimedia Appendix 2 Linear regression of body image distress by age group.

Multimedia Appendix 3 Pearson correlations of body image items with health and well-being measures for each age group.

## Abbreviations

K10: 10-item Kessler Psychological Distress Scale
