# Supplementary material for: Body Image Distress and Its Associations From an International Sample of Men and Women Across the Adult Life Span: Web-Based Survey Study
Source: JMIR Form Res. 2021 Nov 4;5(11):e25329. doi: 10.2196/25329 (PMC8603168; doi:10.2196/25329)
Supplement: Multimedia Appendix 1 [file formative_v5i11e25329_app1.docx]

**Multimedia Appendix 1.**

Frequency statistics, Chi Square and ANOVA comparing body image items by age and sex

|  | **Sex** | | | | **Age-bands (years)** | | | | | | **Full sample** | |
| --- | --- | --- | --- | --- | --- | --- | --- | --- | --- | --- | --- | --- |
|  | **Men** | **Women** | **Statistic** | ***P*** | **16 to 25** | **26 to 49** | **50+** | **Statistic** | ***P*** |  | |  |
| **Body image distress / preoccupation** | 2175 | 3338 | 196.1^a^ | <.001 | 1278 | 1502 | 2733 | 530.7^a^ | <.001 | 5513 | |  |
| ***Yes*** | 39.2 | 58.5 |  |  | 75.2 | 55.7 | 36.9 |  |  | 50.9 | |  |
| ***No*** | 60.8 | 41.5 |  |  | 24.8 | 44.3 | 63.1 |  |  | 49.1 | |  |
| **How much does weight/ shape influence how you think of yourself as a person?** | 2169 | 3327 | 209.8^b^ | <.001 | 1275 | 1497 | 2724 | 114.9^b^ | <.001 | 5496 | |  |
| *1 =* ***Not at al****l* | 23.5 | 12.8 |  |  | 10.2 | 12.1 | 22.9 |  |  | 17.0 | |  |
| *6 =* ***A great deal*** | 11.8 | 23.2 |  |  | 25.9 | 21.4 | 13.8 |  |  | 18.7 | |  |
| *Median score* | 3 | 4 |  |  | 4 | 4 | 3 |  |  | 4 | |  |
| **BMI** | 2115 | 3264 | 42.1^b^ | <.001 | 1248 | 1464 | 2667 | 248.1 | <.001 | 5379 | |  |
| ***Underweight*** | 1.4 | 4.3 |  |  | 7.8 | 2.7 | 1.3 |  |  | 3.2 | |  |
| ***Healthy weight*** | 31.7 | 41.2 |  |  | 57.8 | 39.7 | 26.8 |  |  | 37.5 | |  |
| ***Overweight*** | 37.4 | 25.6 |  |  | 18.6 | 29.9 | 35.9 |  |  | 30.2 | |  |
| ***Obese*** | 29.5 | 28.9 |  |  | 15.9 | 27.7 | 36.0 |  |  | 29.1 | |  |
| **Self-evaluation of weight** | 2176 | 3339 | 27.1 ^b^ | <.001 | 1279 | 1503 | 2733 | 182.7 | <.001 | 5515 | |  |
| ***Very underweight*** | 1.1 | <1.0 |  |  | 1.3 | <1.0 | <1.0 |  |  | <1.0 | |  |
| ***Slightly underweight*** | 7.1 | 4.3 |  |  | 10.1 | 4.9 | 3.4 |  |  | 5.4 | |  |
| ***About the right weight*** | 29.7 | 31.7 |  |  | 44.9 | 34.7 | 22.2 |  |  | 30.9 | |  |
| ***Slightly overweight*** | 46.0 | 40.4 |  |  | 33.5 | 41.5 | 47.4 |  |  | 42.6 | |  |
| ***Very overweight*** | 16.1 | 23.1 |  |  | 10.2 | 18.2 | 26.3 |  |  | 20.4 | |  |
| **Currently dieting** | 2178 | 3339 | 47.1 ^a^ | <.001 | 1278 | 1503 | 2736 | 37.0 ^a^ | <.001 | 5517 | |  |
| ***Yes, to lose weight*** | 19.6 | 26.0 |  |  | 21.0 | 24.9 | 23.9 |  |  | 23.5 | |  |
| ***Yes, to gain weight*** | 2.2 | <1.0 |  |  | 3.0 | 1.1 | 0.8 |  |  | 1.4 | |  |
| ***No*** | 78.1 | 73.2 |  |  | 76.1 | 74.0 | 75.4 |  |  | 75.1 | |  |

^a^ Chi square statistic

^b^ F statistic Analysis of variance (ANOVA)
