# Supplementary material for: Body Image Distress and Its Associations From an International Sample of Men and Women Across the Adult Life Span: Web-Based Survey Study
Source: JMIR Form Res. 2021 Nov 4;5(11):e25329. doi: 10.2196/25329 (PMC8603168; doi:10.2196/25329)
Supplement: Multimedia Appendix 2 [file formative_v5i11e25329_app2.docx]

**Multimedia appendix 2**. Linear regression of body image distress by age group

| **Variable** | ***t*** | ***p*** | **β** | **CI(95%)** | ***F*** | ***df*** | ***P*** | ***Adj. R^2^*** |
| --- | --- | --- | --- | --- | --- | --- | --- | --- |
| **Body image distress or pre-occupation (16 to 25 years)** |  |  |  |  | 12.32 | 14, 1019 | <.001 | 0.13 |
| *Healthy weight (‘no’ vs ‘yes’ based on BMI)* | -1.03 | .30 | -0.03 | [-0.08, 0.03] |  |  |  |  |
| ***Current dieting (‘no’ vs ‘yes’)*** | **3.32** | **.001** | **0.10** | **[0.04, 0.16]** |  |  |  |  |
| ***Perception of weight (‘about the right weight’ vs ‘not’)*** | **5.48** | **<.001** | **0.18** | **[0.10, 0.21]** |  |  |  |  |
| *Physical activity (IPAQ)* | 1.37 | .17 | 0.04 | [-0.01, 0.06] |  |  |  |  |
| ***Psychological distress (K10)*** | **2.23** | **.03** | **0.11** | **[0.00, 0.01]** |  |  |  |  |
| *Suicidal ideation (PSFS)* | -0.29 | .77 | -0.01 | [-0.07, 0.05] |  |  |  |  |
| *Substance misuse* | 0.72 | .47 | 0.02 | [-0.03, 0.06] |  |  |  |  |
| *Days out of role* | 0.24 | .81 | 0.01 | [-0.01, 0.02] |  |  |  |  |
| *Wellbeing (PWI)* | 0.87 | .39 | 0.04 | [-0.00, 0.00] |  |  |  |  |
| ***Happiness (OHQ)*** | **-2.93** | **.003** | **-0.16** | **[-0.00, -0.01]** |  |  |  |  |
| *Resilience (BRCS)* | -0.22 | .83 | -0.01 | [-0.01, 0.01] |  |  |  |  |
| *Social support (SSCS)* | -0.82 | .41 | -0.03 | [-0.02, 0.01] |  |  |  |  |
| *Intimate bonds (IBM)* | -0.21 | .83 | -0.01 | [-0.00, 0.00] |  |  |  |  |
| ***Sex*** | **3.74** | **<.001** | **0.11** | **[0.05, 0.17]** |  |  |  |  |
| **Body image distress or pre-occupation (26 to 45 years)** |  |  |  |  | 15.94 | 14, 1388 | <.001 | 0.13 |
| *Healthy weight (‘no’ vs ‘yes’ based on BMI)* | -0.39 | .70 | -0.01 | [-0.07, 0.05] |  |  |  |  |
| ***Current dieting (‘no’ vs ‘yes’)*** | **5.92** | **<.001** | **0.16** | **[0.12, 0.24]** |  |  |  |  |
| ***Perception of weight (‘about the right weight’ vs ‘not’)*** | **2.86** | **.004** | **0.09** | **[0.03, 0.16]** |  |  |  |  |
| *Physical activity (IPAQ)* | 0.08 | .93 | 0.002 | [-0.03, 0.03] |  |  |  |  |
| ***Psychological distress (K10)*** | **4.78** | **<.001** | **0.20** | **[0.01, 0.02]** |  |  |  |  |
| *Suicidal ideation (PSFS)* | 0.27 | .78 | 0.01 | [-0.05, 0.07] |  |  |  |  |
| ***Substance misuse*** | **2.65** | **.008** | **0.07** | **[0.01, 0.08]** |  |  |  |  |
| *Days out of role* | -1.90 | .06 | -0.05 | [-0.03, 0.00] |  |  |  |  |
| *Wellbeing (PWI)* | -1.32 | .19 | -0.06 | [-0.00, 0.00] |  |  |  |  |
| *Happiness (OHQ)* | -0.34 | .73 | -0.02 | [-0.01, 0.01] |  |  |  |  |
| *Resilience (BRCS)* | -0.52 | .61 | -0.02 | [-0.01, 0.1] |  |  |  |  |
| *Social support (SSCS)* | -0.38 | .71 | -0.01 | [-0.01, 0.01] |  |  |  |  |
| *Intimate bonds (IBM)* | 0.96 | .34 | 0.03 | [-0.00, 0.01] |  |  |  |  |
| ***Sex*** | **3.52** | **<.001** | **0.09** | **[0.04, 0.15]** |  |  |  |  |
| **Body image distress or pre-occupation (50 and over)** |  |  |  |  | 44.27 | 14, 2530 | <.001 | 0.19 |
| *Healthy weight (‘no’ vs ‘yes’based on BMI)* | 1.21 | .23 | 0.03 | [-0.02, 0.08] |  |  |  |  |
| ***Current dieting (‘no’ vs ‘yes’)*** | **7.79** | **<.001** | **0.14** | **[0.12, 0.20]** |  |  |  |  |
| ***Perception of weight (‘about the right weight’ vs ‘not’)*** | **2.72** | **.006** | **0.06** | **[0.02, 0.12]** |  |  |  |  |
| *Physical activity (IPAQ)* | 0.09 | .93 | 0.00 | [-0.02, 0.02] |  |  |  |  |
| ***Psychological distress (K10)*** | **9.41** | **<.001** | **0.27** | **[0.02, 0.02]** |  |  |  |  |
| *Suicidal ideation (PSFS)* | 0.48 | .63 | 0.01 | [-0.04, 0.06] |  |  |  |  |
| *Substance misuse* | 1.86 | .06 | 0.03 | [-0.00, 0.05] |  |  |  |  |
| *Days out of role* | -0.84 | .40 | -0.02 | [-0.01, 0.01] |  |  |  |  |
| ***Wellbeing (PWI)*** | **-3.27** | **.001** | **-0.10** | **[-0.00, -0.00]** |  |  |  |  |
| *Happiness (OHQ)* | 0.31 | .76 | 0.01 | [-0.01, 0.01] |  |  |  |  |
| *Resilience (BRCS)* | 1.75 | .08 | 0.04 | [-0.00, 0.02] |  |  |  |  |
| *Social support (SSCS)* | -1.77 | .08 | -0.04 | [-0.02, 0.00] |  |  |  |  |
| *Intimate bonds (IBM)* | -0.55 | .59 | -0.01 | [-0.00, 0.00] |  |  |  |  |
| ***Sex*** | **8.84** | **<.001** | **0.16** | **[0.12, 0.19]** |  |  |  |  |
