# Supplementary material for: Body Image Distress and Its Associations From an International Sample of Men and Women Across the Adult Life Span: Web-Based Survey Study
Source: JMIR Form Res. 2021 Nov 4;5(11):e25329. doi: 10.2196/25329 (PMC8603168; doi:10.2196/25329)
Supplement: Multimedia Appendix 3 [file formative_v5i11e25329_app3.docx]

Multimedia Appendix 3. Pearson correlations of body image items with health and wellbeing measures for each age group.

| **Item** | **Pearson Correlation (r)** | | | | **N** |
| --- | --- | --- | --- | --- | --- |
|  | **16 to 25** | **26 to 49** | **50+** | **Full sample** |  |
| Weight or shape influence how you think about yourself as a person? (‘no’ *vs ‘*yes’) | 0.50** | 0.54** | 0.53** | 0.55** | 5495 |
| Current dieting status (‘no’ *vs ‘*yes’) | 0.15** | 0.21** | 0.19** | 0.18** | 5511 |
| Self-perception of weight (‘about the right weight’ *vs* all others) | 0.24** | 0.20** | 0.16** | 0.11** | 5513 |
| Healthy weight (‘no’ *vs ‘*yes’ based on BMI) | 0.08^**^ | 0.12** | 0.13** | -0.02 | 5377 |
| Physical Activity (IPAQ) | <.01 | -0.05 | -0.11** | -0.05** | 5513 |
| Psychological distress (K10) | 0.27** | 0.29** | 0.36** | 0.39** | 5513 |
| Suicidal thoughts and behaviours (PSFS) | 0.17** | 0.17** | 0.21** | 0.24** | 5440 |
| Days Out Of Role (BDQ) | 0.14** | 0.10** | 0.13** | 0.14** | 5513 |
| Substance misuse likelihood | 0.07* | 0.13** | 0.07** | 0.07** | 5166 |
| Wellbeing (PWI) | -0.21** | -0.23** | 0.29** | -0.28** | 5513 |
| Happiness (OHS) | -0.27** | -0.23** | 0.27** | -0.30** | 5513 |
| Resilience (BRCS) | -0.17** | -0.15** | -0.15** | -0.20** | 5513 |
| Intimate bonds - care (IBM) | -0.10** | -0.10** | -0.15** | -0.11** | 5513 |
| Social Support (SSCS) | -0.19** | -0.14** | -0.20** | -0.20** | 5513 |

* Correlation is significant at the 0.05 level (2-tailed).

** Correlation is significant at the 0.01 level (2-tailed).
